# Granulin Knock Out Zebrafish Lack Frontotemporal Lobar Degeneration and Neuronal Ceroid Lipofuscinosis Pathology

**DOI:** 10.1371/journal.pone.0118956

**Published:** 2015-03-18

**Authors:** Barbara Solchenberger, Claire Russell, Elisabeth Kremmer, Christian Haass, Bettina Schmid

**Affiliations:** 1 Adolf-Butenandt-Institute—Biochemistry, Ludwig-Maximilians University Munich, Munich, Germany; 2 Department of Comparative Biomedical Sciences, Royal Veterinary College, London, United Kingdom; 3 Institute of Molecular Immunology, Helmholtz Center Munich, Munich, Germany; 4 Munich Cluster for Systems Neurology (SyNergy), Munich, Germany; 5 German Center for Neurodegenerative Diseases (DZNE) Munich, Munich, Germany; Department of Pathology, Anatomy & Cell Biology, Thomas Jefferson University, UNITED STATES

## Abstract

Loss of function mutations in *granulin* (*GRN*) are linked to two distinct neurological disorders, frontotemporal lobar degeneration (FTLD) and neuronal ceroid lipofuscinosis (NCL). It is so far unknown how a complete loss of GRN in NCL and partial loss of GRN in FTLD can result in such distinct diseases. In zebrafish, there are two GRN homologues, Granulin A (Grna) and Granulin B (Grnb). We have generated stable Grna and Grnb loss of function zebrafish mutants by zinc finger nuclease mediated genome editing. Surprisingly, the *grna* and *grnb* single and double mutants display neither spinal motor neuron axonopathies nor a reduced number of myogenic progenitor cells as previously reported for Grna and Grnb knock down embryos. Additionally, *grna^−/−^;grnb^−/−^* double mutants have no obvious FTLD- and NCL-related biochemical and neuropathological phenotypes. Taken together, the Grna and Grnb single and double knock out zebrafish lack any obvious morphological, pathological and biochemical phenotypes. Loss of zebrafish Grna and Grnb might therefore either be fully compensated or only become symptomatic upon additional challenge.

## Introduction

Granulin (GRN) is a pleiotropic growth factor, which plays a role in wound healing, cancer, and inflammation [1]. Heterozygous loss of function mutations in *GRN* are linked to frontotemporal lobar degeneration (FTLD-TDP/*GRN*) [2, 3]. Furthermore, two patients with neuronal ceroid lipofuscinosis (NCL) have been reported to be homozygous for loss of function mutations in *GRN* (NCL/*GRN*) [4]. Neuropathologically, FTLD-TDP/*GRN* patients present with extensive micro- and astrogliosis as well as TAR DNA binding protein 43 (TDP-43) and ubiquitin-positive intracellular inclusions [5–7]. Biochemical studies revealed that lysosomal proteins such as Cathepsin D (CTSD) are increased in brain samples from FTLD-TDP/*GRN* patients [5] suggesting lysosomal dysfunction upon loss of GRN. Moreover, skin biopsies of NCL/*GRN* patients revealed the typical fingerprint profile of lipofuscin aggregates [4]. Grn knock out (KO) mouse models are viable and fertile [1]. Neuropathological examinations of KO mice show also a pronounced micro- and astrogliosis, accumulation of ubiquinated proteins and increased lipofuscinosis [8–13]. Biochemically, *Grn* ^*−/−*^ but not *Grn* ^*+/−*^ mice displayed elevated levels of Ctsd [5, 8, 11], recapitulating features of lysosomal dysfunction. Despite intensive research in the past years, the exact function of GRN and GRN-associated signalling pathways as well as the underlying pathomechanisms in FTLD-TDP/*GRN* and NCL/*GRN* are still elusive. We used zebrafish as a less complex vertebrate model organism with the potential for high throughput drug screening to investigate GRN function in health and disease.

**Fig 1:**
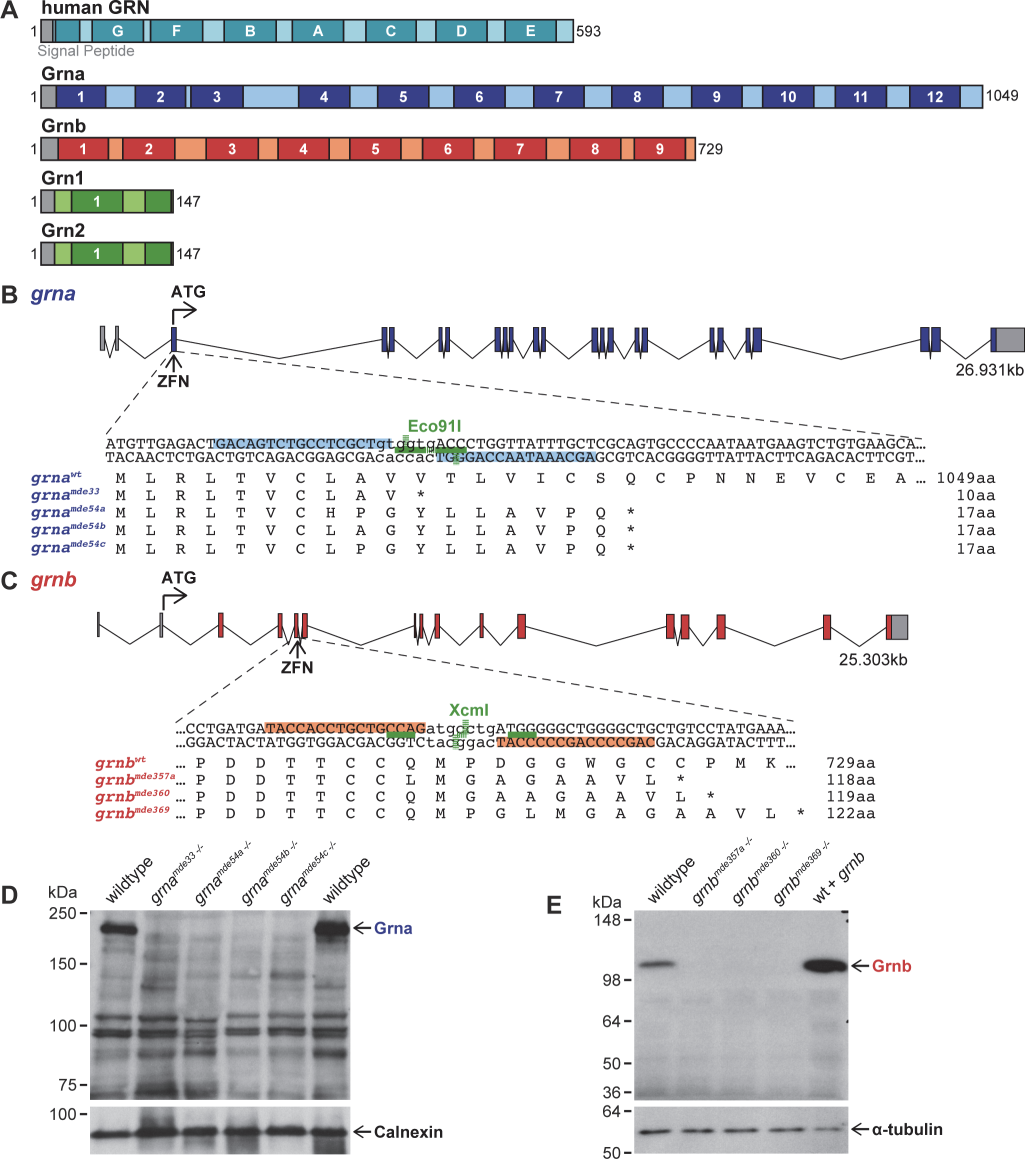
Generation of Grna and Grnb KOs using ZFNs. **A**: Schematic illustration of human GRN and zebrafish Granulins. Human GRN has 7 ½ granulin domains, while 12 granulin domains are found in Grna, 9 in Grnb, and 1 ½ in Grn1 and Grn2. Grey: signal peptide. Black numbers: amino acids. Darker colour and white letters/numbers: granulin domains. **B-C:** Localisation of ZFN target sequences in *grna* and *grnb* and predicted protein sequence of selected alleles. The genomic structure of *grna* and *grnb* is depicted. ZFNs targeting *grna* and *grnb* are located in the first and fourth coding exon, respectively. ZFN-induced genomic lesions in *grna* can be detected with the restriction enzyme (RE) Eco91I and in *grnb* with the RE XcmI. Grey boxes: untranslated region (UTR). Coloured boxes: coding region. Light blue: ZFN binding sites in *grna*. Light red: ZFN binding sites in *grnb*. Green lines: binding sites of the RE. Dashed green line: cut site of the RE. Protein sequences of wildtype (wt) *grna* and 4 *grna* mutation alleles as well as wt *grnb* and 3 *grnb* mutation alleles are shown. *: Stop. **D-E**: Grna and Grnb protein is lost in all mutants. **D**: Grna signal is lost in all adult kidney samples from grna^−/−^ mutants, whereas a signal is present in wt. A Calnexin blot serves as a loading control. **E**: The Grnb signal observed in wt is lost in all 1.5dpf samples from *grnb* ^−/−^ mutants. Injection of *grnb* mRNA leads to an increase in signal. The loading control α-tubulin is present in all samples.

In zebrafish there are two orthologues of *GRN*, *granulin A* (*grna*), *granulin B* (*grnb*) (Fig. 1A). Additionally, zebrafish have two shorter paralogues to *grna* and *grnb* with only one and a half granulin domains referred to as *granulin 1* (*grn1*), and *granulin 2* (*grn2*) [14] (Fig. 1A). Since FTLD-TDP/*GRN* patients have less functional GRN [15–17] and NCL/*GRN* patients have no GRN [4] loss of function models are suitable approaches to mimic aspects of FTLD-TDP/*GRN* and NCL/*GRN*. Grna and Grnb knock down (KD) in zebrafish was previously reported to result in spinal motor neuron (SpMN) axonopathies [18, 19] and a reduced number of myogenic progenitor cells (MPCs) [20]. To obtain stable mutants without phenotypic variability due to the KD procedure, we generated Grna and Grnb KO mutants by genome editing using the zinc finger nuclease (ZFN) technology. Here, we report a genetic, phenotypic and biochemical analysis of Grna and Grnb single and double KO zebrafish.

## Results

### Generation and characterization of Grna and Grnb KO zebrafish

Human and mouse genomes have one *GRN* gene in contrast to the zebrafish genome, which harbours two genes with high homology to mammalian GRN (*grna* and *grnb*) and two short granulins (*grn1* and *grn2*) (Fig. 1A). Grna and Grnb are similar to each other and most similar to mammalian GRN, but are expressed in distinct tissues. At 24 hours post fertilization (hpf) *grna* is most prominently expressed in the intermediate cell mass where precursors of blood and immune cells reside, consistent with the mammalian expression pattern [21], whereas *grnb* is predominantly expressed in various regions of the brain [14]. Grn1 and Grn2 are very short with only one and a half granulin domains (Fig. 1A) and might have a similar function as the proteolytically processed GRN peptides in mammals. We therefore choose Grna and Grnb for the GRN loss of function analysis in zebrafish. Targeted genome editing was performed using ZFNs. The *grna* ZFNs target the first coding exon of *grna* (Fig. 1B), and the *grnb* ZFNs the fourth coding exon of *grnb* (Fig. 1C). ZFN mRNAs were injected at one-cell stage. The embryos were raised to adulthood (P0 generation) and then outcrossed to wildtype zebrafish. The resulting F1 generation was screened for successful germline transmission of induced mutations. The efficiency of ZFNs to introduce mutations in the germline was 75% (27/36 P0 fish) for *grna* and 62% (29/47 P0 fish) for *grnb*. The adult F1 generation was further analysed for ZFN-induced genomic lesions by sequencing.

Offspring from 13 *grna* and 8 *grnb* P0 founder fish were analysed for induced mutations and we detected up to 5 and 3 distinct mutations originating from a single founder fish, respectively. In total 13 different mutations (deletions, insertion and indels) were isolated for *grna* of which 6 result in a frameshift in the coding sequence. For *grnb* 15 different mutations were isolated of which 11 lead to a frameshift in the coding sequence. 4 *grna* and 3 *grnb* frameshift mutations that result in a premature translation termination codon were selected for further characterization (Fig. 1B, C). mRNAs containing a premature translation termination codon are known subjects to nonsense-mediated mRNA decay (NMD), one of the cells endogenous quality control mechanisms that prevents the synthesis of truncated, non-functional proteins [22]. In all *grna* ^*−/−*^ mutants analysed this results in approx. 50% reduction of *grna* mRNAs and in *grnb* ^*−/−*^ mutants the *grnb* mRNA levels were reduced by approx. 90% (S1 Fig.). The differences in residual mRNA levels might be attributed to the presence of a *grna* antisense RNA [14], which potentially prevents the efficient degradation of mutated *grna* by NMD. We generated monoclonal antibodies specifically detecting zebrafish Grna or Grnb protein to examine if the *grna* ^*−/−*^ and *grnb* ^*−/−*^ mutants are deficient in Grna and Grnb, respectively. Neither Grna nor Grnb was detectable in *grna* ^*−/−*^ and *grnb* ^*−/−*^ mutants (Fig. 1D, E), indicating that no protein is made in homozygous mutants.

### Analysis of Grna and Grnb KOs for morphological phenotypes

Morphologically, the *grna* ^*−/−*^ and *grnb* ^*−/−*^ as well as the *grna* ^*−/−*^;*grnb* ^*−/−*^ double mutant embryos and adults are indistinguishable from wildtype embryos (S2 Fig.). Incrosses of *grna* ^*+/−*^, *grnb* ^*+/*^,^-^ and *grna* ^*+/−*^;*grnb* ^*+/−*^ adults produce offspring of all genotypes in an expected Mendelian ratio. Also offspring from incrosses of *grna* ^*−/−*^, *grnb* ^*−/−*^, and *grna* ^*−/−*^;*grnb* ^*−/−*^ adults had normal amounts of fertilized embryos and no morphological defects (data not shown).

Since GRN is a growth factor, we asked if the development is delayed in single and double homozygous *grna* and *grnb* mutant embryos. The developmental stage of the mutants was assessed by counting somites at a time point when the controls embryos reached the 13–15 somite stage. No developmental delay was detected in *grna* ^*−/−*^, *grnb* ^*−/−*^, and *grna* ^*−/−*^;*grnb* ^*−/−*^ mutants when compared to wildtype embryos (S3 Fig.). As there are four Grn in zebrafish, we speculated, that the paralogues could compensate for each other by upregulation. Therefore, the *grnb* and *grna* mRNA expression levels and protein levels were determined in Grna and Grnb KO larvae, respectively. Additionally, the *grn1*/*grn2* mRNA expression levels were analysed in *grna* ^*−/−*^;*grnb* ^*−/−*^ larvae. No compensatory upregulation of the *grna* or *grnb* paralogue on mRNA and protein level was detected in *grna* ^*−/−*^ or *grnb* ^*−/−*^ mutants, respectively (S4 Fig. A-B,D-E). Moreover, the *grna* ^*−/−*^;*grnb* ^*−/−*^ mutants did not show any transcriptional upregulation of *grn1/grn2* (S4 Fig. 4C).

In summary, homozygous Grna and Grnb single and double KO mutants were successfully generated. They have wildtype morphology, are viable and fertile and are not developmentally delayed. Moreover, lack of Grna or Grnb in zebrafish does not result in compensatory transcriptional or translational upregulation of Grnb or Grna, respectively.

### Analysis of SpMN in Grna and Grnb KOs

**Fig 2:**
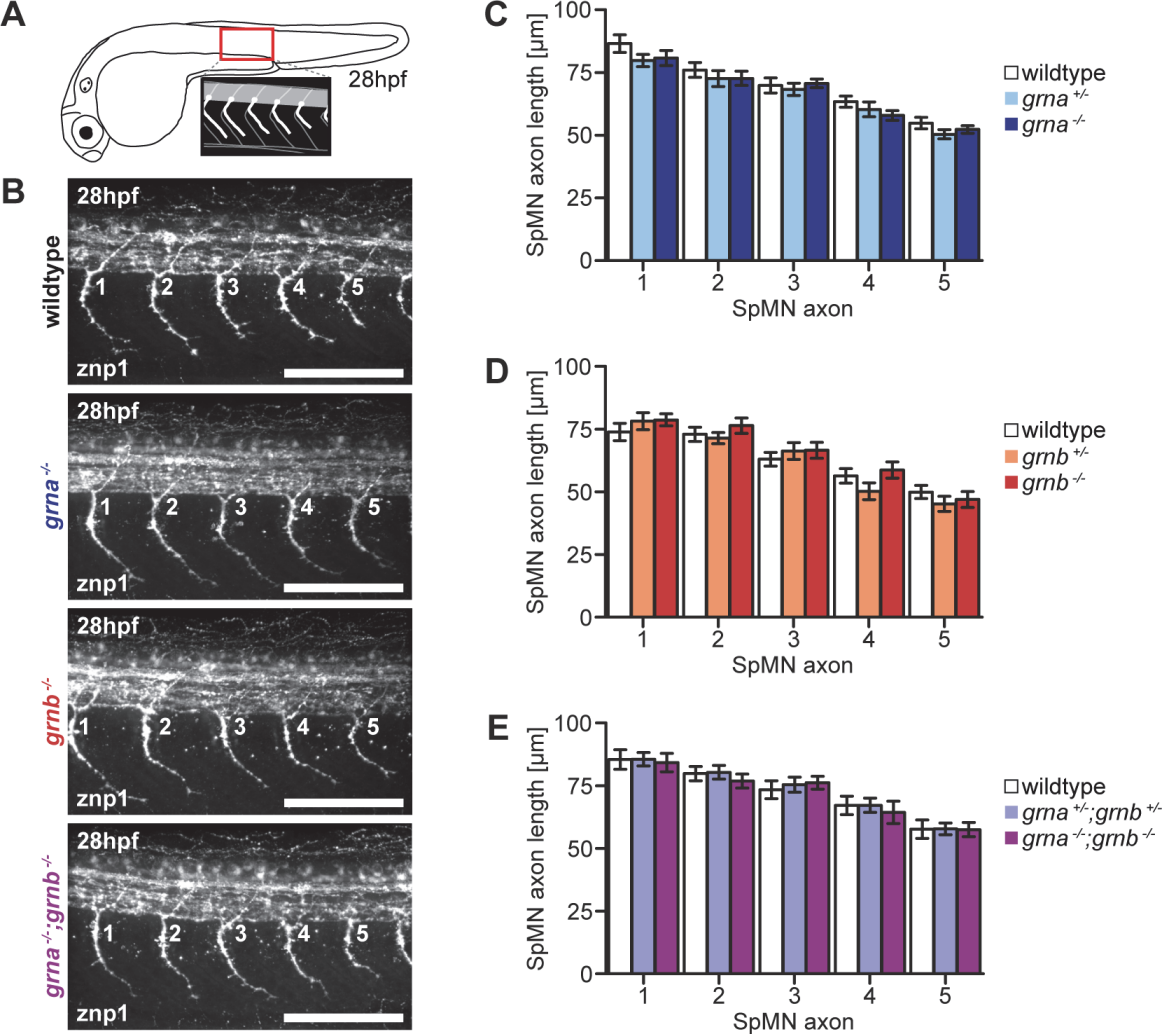
No SpMN axonopathy in Grna and Grnb single and double KOs. **A**: Schematic illustration of a zebrafish embryo at 28hpf (lateral view) and a detail of the region above the end of the yolk extension imaged for the analysis of SpMN axons (lateral view). **B:** In Grna and Grnb single and double KOs the SpMN axons show no extended branching. Whole-mount immunofluorescence staining of 28hpf embryos with znp1 antibody. The 5 SpMN axons above the end of the yolk extension are shown. Images taken by spinning disk confocal microscopy. Anterior to the left. Lateral view. Orthogonal projections. Scale bar: 100μm. **C-E**: Quantification of the SpMN axon length in homozygous and heterozygous Grna and Grnb single and double KOs and wildtype siblings. The SpMN axon length of the 5 SpMN axons (1–5) above the end of the yolk extension is measured from the exit point of the spinal cord to the tip of the growth cone. **C**: Homozygous and heterozygous Grna KOs and wildtype siblings. n = 30. **D**: Homozygous and heterozygous Grnb KOs and wildtype siblings. n = 30. **E**: Homozygous and heterozygous Grna and Grnb KOs and wildtype siblings. n = 25. S.E.M. Two-way ANOVA. Bonferroni post-test. All non-significant (n.s.).

SpMN axons from Grna and Grnb KD embryos using MOs were reported to be truncated and hyperbranched [18, 19, 23]. We therefore stained SpMN axons of single and double Grna and Grnb KO embryos at 28hpf with the znp1 antibody. We previously experienced that variations between different clutches can result in slight differences in the SpMN axon length. Therefore, we only compared embryos from the same clutch to avoid inter-clutch variation and analysed the five SpMN axons anterior to the end of the yolk extension (Fig. 2A) [24, 25]. First, wildtype, heterozygous and homozygous embryos from matings of single and double heterozygous Grna and Grnb KOs were compared. Extended branching was not observed in any of the genotypes analysed (Fig. 2B). Measurements of the SpMN axon length, which is defined here as the length of the SpMN axon from the exit point of the spinal cord to the tip of the growth cone, revealed that the length is not significantly altered in any genotype analysed (Fig. 2C-E). When translation inhibition MOs are used for KD experiments maternal and zygotic mRNA is depleted whereas in KO animals from heterozygous females maternal mRNA could potentially preclude a phenotype. To exclude that maternal mRNA precludes SpMN axon outgrowth phenotypes, siblings from matings of homozygous females and heterozygous males were analysed. Also in maternal zygotic KOs of Grna, Grnb, or both, we did not detect any extended branching or reduced SpMN axon outgrowth (S5 Fig.).

In summary, SpMN axons and their length are indistinguishable in single and double Grna and Grnb stable genetic KO, in contrast to previously published reports from Grna and Grnb KD experiments [18, 19, 23].

### Analysis of MPCs in Grna and Grnb KOs

**Fig 3:**
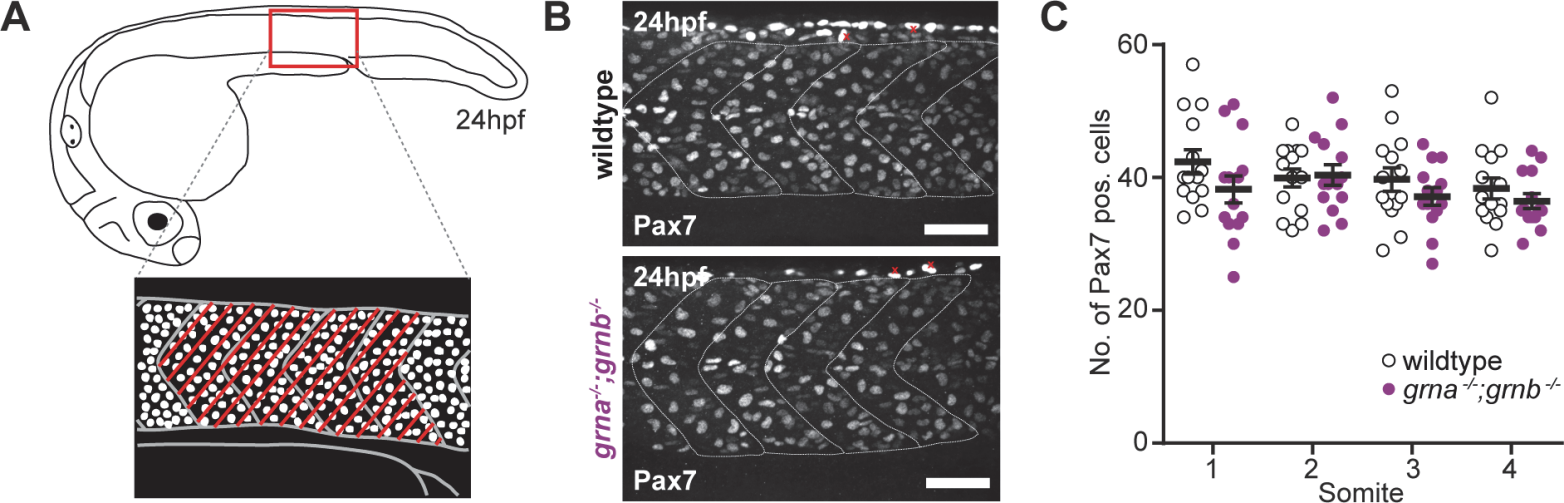
The number of MPCs is equal. **A**: Schematic illustration of a zebrafish embryo at 24hpf (lateral view) and a detail of the region above the end of the yolk extension imaged for the analysis of the MPCs (lateral view). The four somites, which were considered for the quantification of MPCs are marked with red lines. **B**: Immunofluorescence staining with Pax7 at 24hpf in *grna* ^*−/−*^;*grnb* ^*−/−*^ mutants and wildtype embryos. In each image two xanthophores are exemplarily marked with a red x. Images taken by spinning disk confocal microscopy. Anterior to the left. Lateral view. Orthogonal projections. Scale bar: 50μm. **C**: Quantification of Pax7-positive cells in the 4 somites (1–4) above the end of the yolk extension in *grna* ^*−/−*^;*grnb* ^*−/−*^ mutants and wildtype embryos. Only Pax7-positive cells at the surface of the somites were counted. n = 15. S.E.M. Mann-Whitney test (two-tailed). All n.s. S1: p = 0.0854. S2: p = 0.7977. S3: p = 0.3489. S4: p = 0.2337.

A reduced number of MPCs was reported in Grna KD embryos [20]. A similar reduction, although less strong, was also reported for Grnb KD [20]. We performed immunofluorescence staining with the Pax7 antibody in wildtype and *grna* ^*−/−*^;*grnb* ^*−/−*^ embryos at 24hpf and quantified the number of Pax7-positive cells in the four somites anterior to the end of the yolk extension (Fig. 3A). At this stage of development Pax7 labels the nuclei of MPCs, which are located at the surface of the trunk musculature [26] but also some xanthophores [27]. Xanthophores can be distinguished from Pax7-positive MPCs by their elongated shape, a more intense staining as well as the dorsal localization [27]. Images of Pax7-stained wildtype and *grna* ^*−/−*^;*grnb* ^*−/−*^ embryos do not reveal any differences. In both, wildtype and *grna* ^*−/−*^;*grnb* ^*−/−*^ embryos, nuclei are stained with the Pax7 antibody in different intensities and a few of them show an elongated shape (Fig. 3B). Quantification of the number of Pax7-positive nuclei in the most lateral part of the four somites anterior to the end of the yolk extension, independent of their staining intensity, revealed that the number of Pax7-positive cells is not significantly decreased in all four somites analysed of *grna* ^*−/−*^;*grnb* ^*−/−*^ mutants when compared to wildtype embryos (Fig. 3C). Taken together, there is no significant reduction in Pax7-positive MPCs in homozygous Grna;Grnb KO embryos.

### Analysis of Grna and Grnb KOs for disease-related pathology

**Fig 4:**
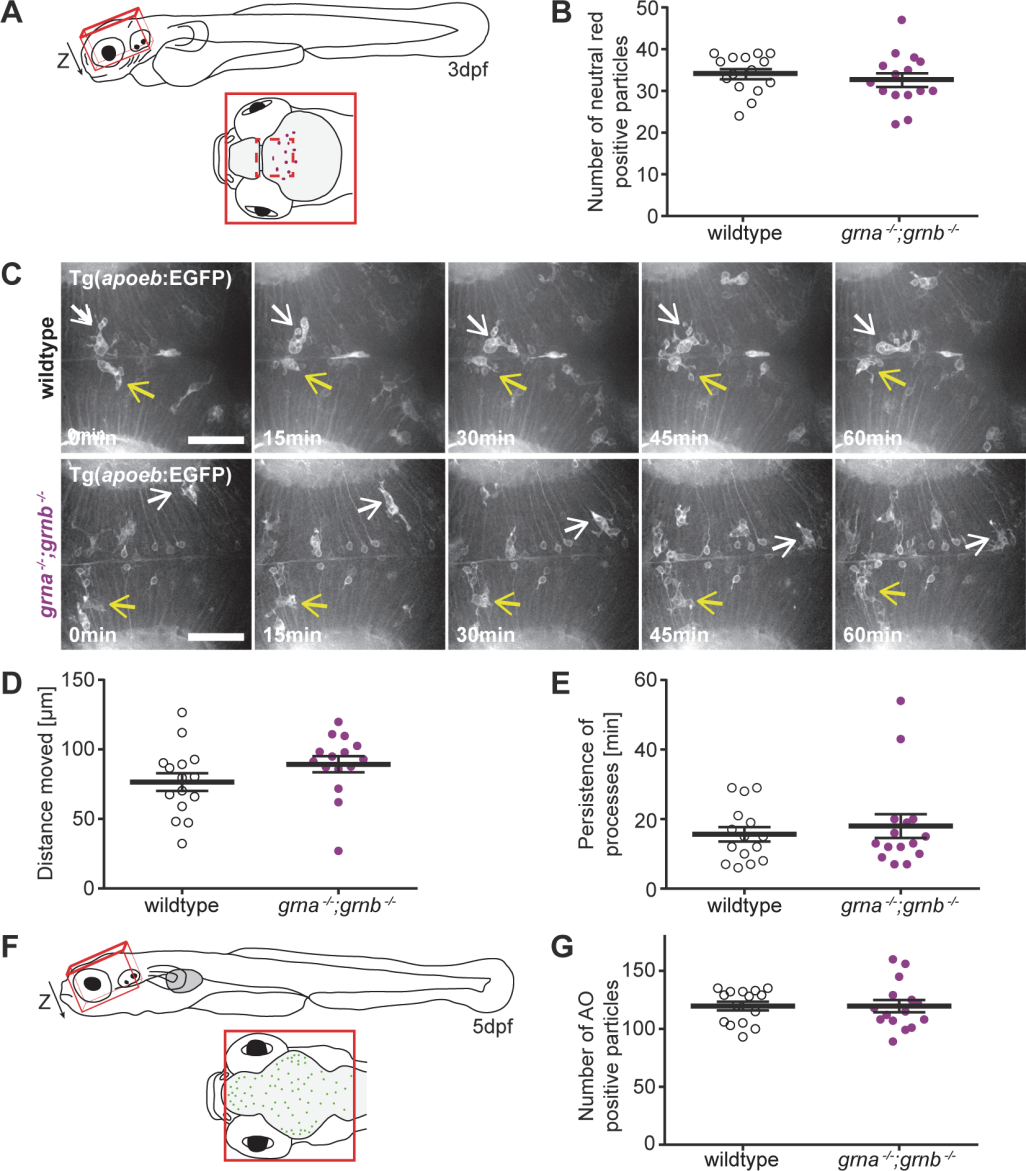
No microgliosis and neurodegeneration in *grna* ^*−/−*^;*grnb* ^*−/−*^ mutants. **A**: Schematic illustration of a zebrafish larvae at 3dpf (lateral view) and a detail of the region (red line), dorsal view, imaged for the analysis of neutral red positive particles (B). The dashed red line marks the area that was imaged in the time lapse recordings of microglia (C). **B**: The number of neutral red positive particle in the region illustrated in A (Z-stack) is unchanged in wildtype and *grna* ^*−/−*^;*grnb* ^*−/−*^ mutants. n = 15. S.E.M. Mann-Whitney test (two-tailed). p = 0.2884. **C-E**: Microglia in Tg(*apoeb*:lynEGFP) *grna* ^*−/−*^;*grnb* ^*−/−*^ mutants and wildtype larvae at 3dpf are indistinguishable. **C:** Still images of the time lapse recordings in the optic tectum recorded by spinning disk confocal microscopy. Two microglia cells marked in each genotype by a white and yellow arrow. Dorsal view. Anterior to the left. n = 3. Scale bar: 50μm. Recording time: 60min. 1frame/min. **D:** The distance microglia move within one hour in *grna* ^*−/−*^;*grnb* ^*−/−*^ mutants and wildtype larvae. Quantification of n = 3x5 randomly selected microglia from the time lapse recordings shown in C. S.E.M. Mann-Whitney test (two-tailed). p = 0.0671. **E:** Processes in the *grna* ^*−/−*^;*grnb* ^*−/−*^ mutants and wildtype larvae persist for same durations. Quantification of n = 3x5 randomly selected processes from the time lapse recordings shown in C. S.E.M. Mann-Whitney test (two-tailed). p = 0.8296. **F:** Schematic illustration of a zebrafish larvae at 5dpf (lateral view) and a detail of the region, dorsal view, imaged for the analysis of acridine orange (AO) positive cells. **G**: The number of acridine orange positive cells in the region illustrated in C (Z-stack) is unchanged in wildtype and *grna* ^*−/−*^;*grnb* ^*−/−*^ mutants. n = 15. S.E.M. Mann-Whitney test (two-tailed). p = 0.69.

FTLD-TDP/*GRN* patients [28–30] and homozygous Grn KO mice [8–13] develop micro- and astrogliosis. Moreover, *grna* is expressed in microglia and has even been used as a microglia marker [31, 32]. We therefore asked, if *grna* ^*−/−*^;*grnb* ^*−/−*^ mutants develop brain gliosis. To estimate the number of microglia in the larval brain at 3dpf we stained with neutral red, a dye that accumulates as red fluorescence in acidic vesicles of microglia and macrophages [33], and quantified the number of neutral red positive particles in the brain (Fig. 4A). This analysis did not reveal any differences in the amount of neutral red particles in the brain. We conclude that the number of microglia is not increased in the brain of *grna* ^*−/−*^;*grnb* ^*−/−*^ mutants when compared to wildtype at 3dpf (Fig. 4B). Microglia in the larval brain are highly motile and phagocytic and mature to ramified microglia with small cell bodies from 5dpf onward [34]. Therefore, it is not feasible to categorize microglia in the larval zebrafish brain in active and resting microglia. A time lapse analysis of amoeboid microglia, using a transgenic line that labels a subset of microglia, revealed that the distance moved and the persistence of processes is identical in *grna* ^*−/−*^;*grnb* ^*−/−*^ and wildtype larvae at 3dpf (Fig. 4C-E) indicating that *grna* ^*−/−*^;*grnb* ^*−/−*^ mutants seem to lack a morphological microglial phenotype at larval stages.

**Fig 5:**
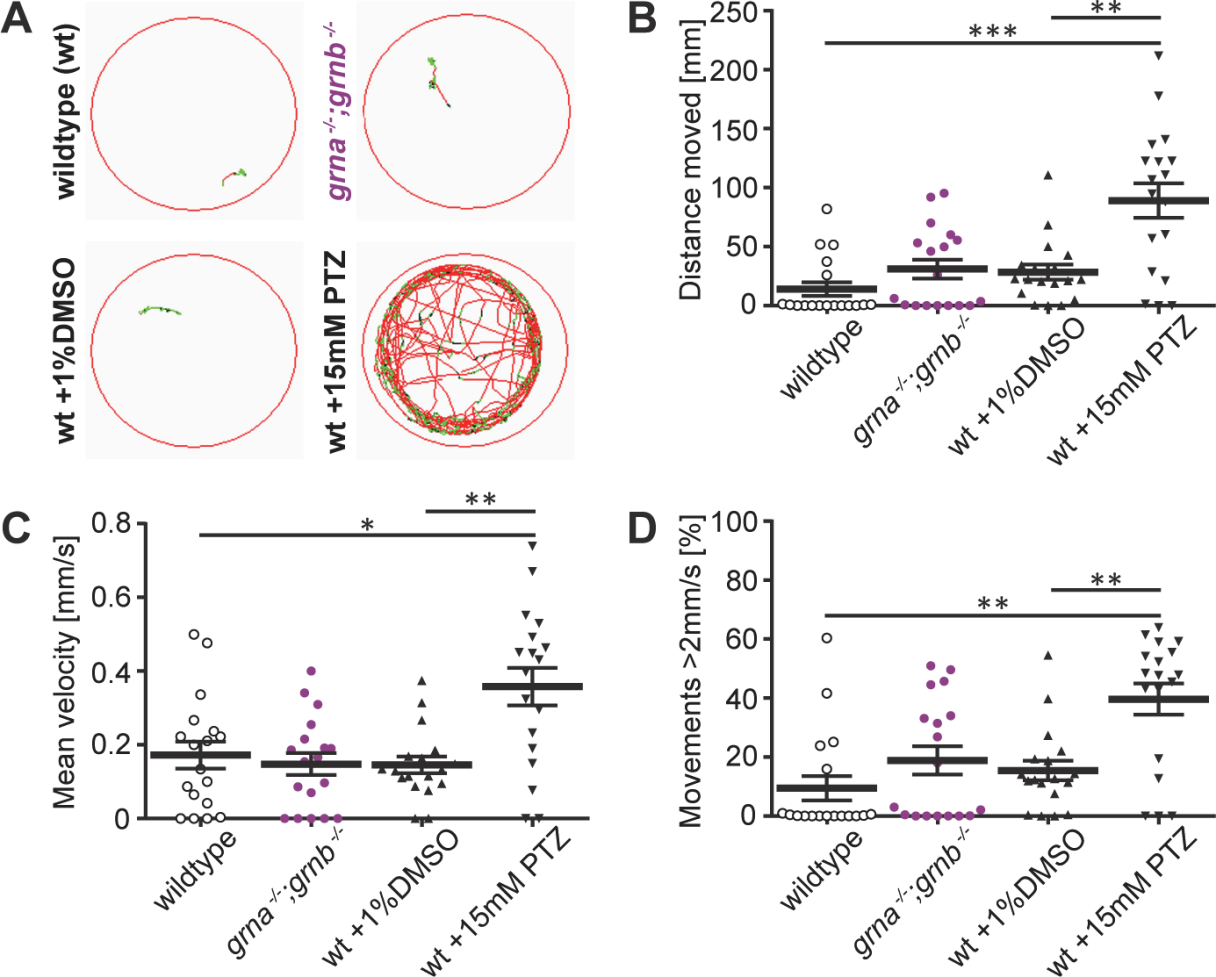
*grna* ^*−/−*^;*grnb* ^*−/−*^ mutants swim like wildtype. **A**: The swim path of wildtype, *grna* ^*−/−*^;*grnb* ^*−/−*^ mutants, DMSO-treated, and PTZ-treated larvae is shown. PTZ treatment was used as a positive control. 5dpf. Movements < 2mm/s: black lines. Movements 2–6mm/s: green lines. Movements > 6mm/s: red lines. Recording time: 5min. **B**: The total distance moved within 5min in wildtype, *grna* ^*−/−*^;*grnb* ^*−/−*^ mutants, DMSO-treated, and PTZ-treated larvae is shown. Wt-*grna* ^*−/−*^;*grnb* ^*−/−*^: p = 0.2386. Wt-DMSO: p = 0.0534. Wt-PTZ: ***p = 0.0002. DMSO-PTZ: **p = 0.004. **C**: A graph of the mean velocity of wildtype, *grna* ^*−/−*^;*grnb* ^*−/−*^ mutants, DMSO-treated, and PTZ-treated larvae is shown. Time frame: 5min. Wt-*grna* ^*−/−*^;*grnb* ^*−/−*^: p = 0.5657. Wt-DMSO: p = 0.8081. Wt-PTZ: *p = 0.0137. DMSO-PTZ: **p = 0.0014. **D**: Percentage of time spent for movements with a velocity above 2mm/s in wildtype, *grna* ^*−/−*^;*grnb* ^*−/−*^ mutants, DMSO-treated, and PTZ-treated larvae is plotted. Time frame: 5min. Wt-*grna* ^*−/−*^;*grnb* ^*−/−*^: p = 0.2585. Wt-DMSO: p = 0.0668. Wt-PTZ: **p = 0.0016. DMSO-PTZ: **p = 0.0037. **B-D:** n = 18. S.E.M. Mann-Whitney test (two-tailed).

Smith and colleagues linked *GRN* loss of function to NCL [4]. Furthermore, Grn KO mice develop NCL-related pathological and biochemical alterations [5, 8, 10, 11, 35]. We therefore analysed the *grna* ^*−/−*^;*grnb* ^*−/−*^ mutants for NCL-related behavioural, pathological, and biochemical alterations. Due to massive neurodegeneration one of the clinical symptoms described in the NCL/*GRN* patients is epilepsy [4]. We first examined if cell death is increased in the larval brain of *grna* ^*−/−*^;*grnb* ^*−/−*^ mutants at 5dpf by acridine orange staining, a vital dye that is frequently used in zebrafish to estimate the amount of cell death [25]. The number of acridine orange positive particles was quantified in the area of the larval brain marked by a square in the illustration (Fig. 4F). This quantification revealed that there is no increase in acridine orange positive particles in the *grna* ^*−/−*^;*grnb* ^*−/−*^ mutants when compared to age-matched wildtype embryos (Fig. 4G). Despite the lack of neurodegeneration in the *grna* ^*−/−*^;*grnb* ^*−/−*^ mutants, we tracked the swim path in *grna* ^*−/−*^;*grnb* ^*−/−*^ mutants, potentially revealing seizure-like swim behaviour, which has been described in a CLN2-NCL disease model zebrafish [36]. We analysed the swimming behaviour of 5dpf *grna* ^*−/−*^;*grnb* ^*−/−*^ mutants and age matched wildtype larvae for 5min after an adaptation period of 30min in the recording device and plotted the swim path. Moreover, we extracted the total distance moved, the mean velocity and the percentage of time spent for movements with a velocity above 2mm/s from the data sets acquired. Seizure-like swim behaviour in zebrafish has been described as quick, circling spinning [36]. As a positive control we applied pentylenetetrazole (PTZ), a drug that was previously demonstrated to induce seizures in zebrafish [37]. In contrast to wildtype siblings and *grna* ^*−/−*^;*grnb* ^*−/−*^ mutants, the PTZ-treated larvae are much more active, which is reflected in the total distance moved (Fig. 5A,B). The mean velocity as well as the percentage of time spent for movements faster than 2mm/s is comparable in wildtype and *grna* ^*−/−*^;*grnb* ^*−/−*^ mutants but significantly increased in the PTZ-treated wildtype larvae (Fig. 5C,D). This data indicate that swimming of 5dpf *grna* ^*−/−*^;*grnb* ^*−/−*^ mutants is very similar to that of wildtype larvae and devoid of seizure-like behaviour as seen upon PTZ treatment.

**Fig 6:**
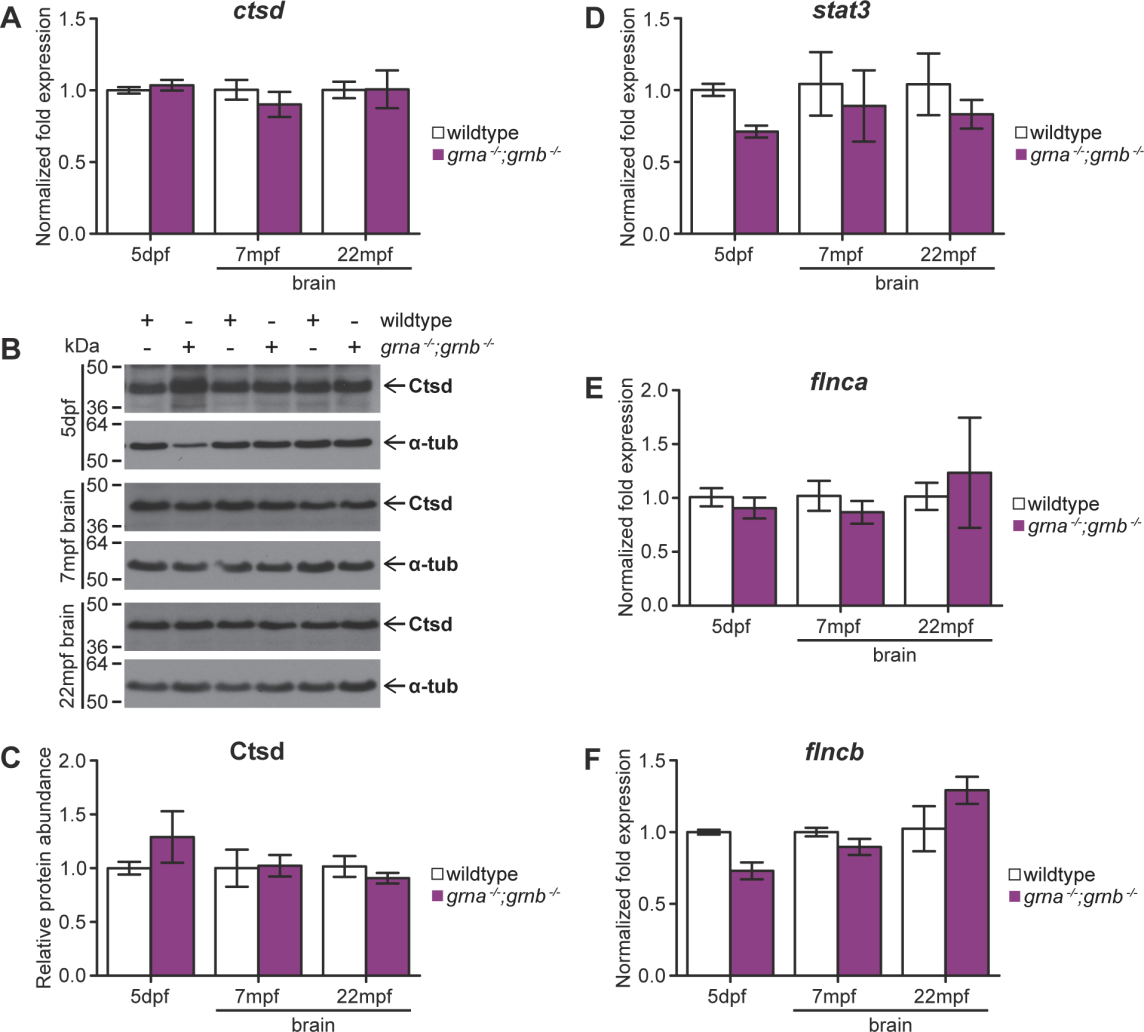
No disease-related biochemical alterations in *grna* ^*−/−*^;*grnb* ^*−/−*^ mutants. **A-C**: *ctsd* mRNA expression and Ctsd protein levels in *grna* ^*−/−*^;*grnb* ^*−/−*^ mutants. **A**: *ctsd* mRNA expression at 5dpf and in 7mpf as well as 22mpf brain samples derived from *grna* ^*−/−*^;*grnb* ^*−/−*^ mutants compared to wildtype controls. 5dpf: p = 1.0. 7mpf: p = 0.4. 22mpf: p = 0.7. **B**: Western blots showing Ctsd and α-tubulin in 5dpf whole lysis samples as well as 7mpf and 22mpf brain samples from *grna* ^*−/−*^;*grnb* ^*−/−*^ mutants and wildtype controls. **C**: Quantification of Ctsd from the Western blots shown in B normalized to α-tubulin. 5dpf: n = 4. 7mpf and 22mpf: n = 3. 5dpf: p = 0.8857. 7mpf: p = 1.0. 22mpf: p = 0.7. **D**: *stat3* mRNA levels in 5dpf *grna* ^*−/−*^;*grnb* ^*−/−*^ mutants and 7mpf as well as 22mpf *grna* ^*−/−*^;*grnb* ^*−/−*^ mutant brains compared to wildtype control samples. 5dpf: p = 0.1. 7mpf: p = 0.4. 22mpf: p = 0.4. **E**: *flnca* mRNA levels in *grna* ^*−/−*^;*grnb* ^*−/−*^ larvae at 5dpf and 7mpf as well as 22mpf *grna* ^*−/−*^;*grnb* ^*−/−*^ brain samples in comparison to wildtype samples. 5dpf: p = 0.4. 7mpf: p = 0.7. 22mpf: p = 0.7. **F**: *grna* ^*−/−*^;*grnb* ^*−/−*^ larvae of 5dpf and 7mpf and 22mpf *grna* ^*−/−*^;*grnb* ^*−/−*^ brain samples are analysed for *flncb* mRNA expression levels and compared to wildtype. 5dpf: p = 0.1. 7mpf: p = 0.4. 22mpf: p = 0.4. A,D-E: Normalized to *actb1* and *tbp*. qPCR. 5dpf: n = 4. 7mpf and 22mpf: n = 3. A,C-E: S.E.M. Mann-Whitney test (two-tailed).

To address, if the lysosomal function is altered as in NCL patients lacking GRN, we determined the expression of Ctsd on the transcriptional and translational level. Grn KO mice have elevated *Ctsd* mRNA expression and Ctsd protein levels [5, 11, 38]. Moreover, FTLD-TDP/*GRN* patients have increased CTSD levels in the frontal cortex [5]. In contrast to mice and human, the *ctsd* mRNA expression levels were unchanged in samples derived from 5dpf *grna* ^*−/−*^;*grnb* ^*−/−*^ mutants and adult *grna* ^*−/−*^;*grnb* ^*−/−*^ brains of up to 22-month-old zebrafish, when compared to the corresponding wildtype controls (Fig. 6A). Ctsd immuno-blots of 5dpf larvae and adult brain samples showed no difference on the Ctsd protein level between *grna* ^*−/−*^;*grnb* ^*−/−*^ mutants and age-matched wildtype controls (Fig. 6B,C).

A quantitative proteomic analysis of the zebrafish TDP-43 orthologous (*tardbp* ^*−/−*^;*tardbpl* ^*−/−*^) identified Flnca and Stat3 to be upregulated upon loss of Tardbp;Tardbpl [24]. Interestingly TDP-43 aggregates are a hallmark of FTLD-TDP/*GRN* and also in brain samples from FTLD patients *FLNC* mRNA expression levels are significantly increased [24]. In contrast, qPCR experiments revealed a slight but not statistically significant decrease in *stat3* expression in samples derived from *grna* ^*−/−*^;*grnb* ^*−/−*^ mutants, when compared to wildtype controls (Fig. 6D). qPCR analysis of the two *FLNC* orthologous in zebrafish, *flnca* and *flncb*, revealed that *flnca* mRNA expression levels are not altered in *grna* ^*−/−*^;*grnb* ^*−/−*^ mutants and wildtype controls (Fig. 6E). mRNA expression of *flncb* appears to be decreased in 5dpf *grna* ^*−/−*^;*grnb* ^*−/−*^ mutants when compared to wildtype control larvae, but this is not statistically significant. *flncb* is expressed at comparable levels in 7mpf *grna* ^*−/−*^;*grnb* ^*−/−*^ and control brains and slightly but not significantly increased at 22mpf in *grna* ^*−/−*^;*grnb* ^*−/−*^ brains when compared to control brains (Fig. 6F).

In summary, neuropathology as well as biochemical alterations reported in Grn-deficient humans and mice and the TDP-43 loss of function zebrafish were not observed in *grna* ^*−/−*^;*grnb* ^*−/−*^ mutant zebrafish.

## Discussion

### Grna and Grnb mutants are complete loss of function mutants

Among the four zebrafish *granulins*, *grna* and *grnb* were chosen for the generation of KO mutants since they have a domain structure reminiscent of mammalian GRN. *grna* and *grnb* are both orthologous to human GRN with potentially redundant functions [14, 17]. Interestingly, we isolated a *grna* transcript with 12 granulin domains instead of the 10 granulin domains previously described [14]. Also in contrast to previous publications, Grna and Grnb [18, 20] were migrating at a higher molecular weight of approx. 210–230kDa and approx. 100–120kDa in denaturing SDS-PAGE.

The homozygous Grna and Grnb single and double KOs are viable and show no obvious morphological phenotype and fertility differences. Moreover, the lack of Grna and Grnb does not slow development.

### No FTLD-TDP/*GRN*- and NCL/*GRN*-related phenotypes in *grna* ^*−/−*^;*grnb* ^*−/−*^ mutants

Among the most consistent neuropathological findings in Grn KO mice are increased micro- and astrogliosis and enhanced lipofuscinosis [1] both neuropathological hallmarks of FTLD-TDP/*GRN* and NCL/*GRN* patients, respectively [4, 28–30]. In zebrafish larvae amoeboid microglia, which are highly motile and phagocytic, are present already at 3dpf and phagocytise cells and debris during brain development in a healthy environment [33, 34, 39]. These larval microglia, which can respond to inflammatory stimuli [40], are indistinguishable in a motion analysis performed with microglia of Grna;Grnb KO and wildtype larvae. A phenotypic categorization of amoeboid microglia according to the terms active and resting, commonly used for the analysis of microglia in the adult central nervous system [41] does not apply in larval zebrafish [34]. As the number of microglia in the Grna;Grnb KO larvae is also not increased we concluded that the *grna* ^*−/−*^;*grnb* ^*−/−*^ mutants are devoid of microgliosis. “Astrocytes” are different in zebrafish compared to mouse. Zebrafish radial ependymoglia located at the ventricle fulfil functions attributed to parenchymal astrocytes in mammals [41], indicating that the cell types involved in inflammation in zebrafish are different than in mammals. Even though Grn KO mice develop micro- and astrogliosis they do not suffer from neurodegeneration suggesting that micro- and astrogliosis is not a result of the neurodegeneration in patients. To our knowledge, there is so far no zebrafish model for gliosis in the absence of injury. Therefore, it remains to be shown if this pathology can occur in zebrafish at all. In wildtype zebrafish the existence of autofluorescent neurolipofuscin is controversially discussed [42, 43]. Our attempts to demonstrate neurolipofuscin in aged wildtype brains using PAS stain supports the absence of lipofuscin deposits in aged zebrafish [42, 44].

In contrast to a CLN2 zebrafish NCL disease model associated with mutations in tripeptidyl peptidase 1 (TPP1) [36], *grna* ^*−/−*^;*grnb* ^*−/−*^ mutants are devoid of increased cell death in the brain and do not display movement phenotypes reminiscent of seizures, a clinical hallmark of NCL pathology [36]. Neurodegeneration was only reported in one aged Grn KO mouse strain [8] but not in others [9, 11] consequently seizures are also not likely to occur in Grn KO mice. Possibly, seizures are specific for the clinical representation in humans due to the massive neurodegeneration observed in the NCL/*GRN* patients [4].

Biochemical alterations observed in FTLD-TDP/*GRN* and Grn KO mice [5, 11, 24] were not detectable in *grna* ^*−/−*^;*grnb* ^*−/−*^ mutant zebrafish. The lysosomal protease Ctsd, which is itself linked to NCL by disease causing loss of function mutations [45], is slightly different in zebrafish compared to mammalian CTSD. Zebrafish Ctsd is mono-glycosylated and matures into a single-chain protein, whereas the di-glycosylated human CTSD proprotein is cleaved into a light and heavy chain [46, 47]. However, both the proprotein and the heavy chain are increased in Grn KO mice [5], whereas zebrafish Ctsd is unchanged. In Tardbp/Tardbpl-deficient zebrafish Stat3 was increased, whereas there was a trend to slightly but not significantly decreased *stat3* expression in *grna* ^*−/−*^;*grnb* ^*−/−*^ mutants. Whether *STAT3* is increase in FTLD-TDP/*GRN* and Grn KO mice is not examined so far. *STAT3* is involved in many processes, including neuroinflammation [48]. Therefore, it is possible, that a *STAT3* increase occurs as a result of micro- and astrogliosis observed in FTLD-TDP/*GRN* and Grn KO mice [8–13]. If this is the case it would explain why *stat3* is not increased in *grna* ^*−/−*^;*grnb* ^*−/−*^ mutant zebrafish as they are devoid of microgliosis. The *flnca* and *flncb* expression levels are not significantly increased in *grna* ^*−/−*^;*grnb* ^*−/−*^ mutants. It is possible, that the *grna* ^*−/−*^;*grnb* ^*−/−*^ mutant zebrafish were too young when they were analysed, as the pathology and biochemical alterations do also manifest themselves over time in the Grn KO mice [1, 5]. Furthermore, heterozygous Grn KO mice did not develop micro- and astrogliosis and lack biochemical signatures described in the homozygous Grn KO mice [8, 35].

### Potential reasons for the absence of phenotypes in *grna* ^*−/−*^;*grnb* ^*−/−*^ mutants

We hypothesize that additional challenges are needed in the *grna* ^*−/−*^;*grnb* ^*−/−*^ mutants to obtain disease-related phenotypes. In line with this hypothesis is the observation that the disease onset and manifestation in FTLD-TDP/*GRN* patients is quite variable [49, 50] supporting the need of a second hit such as oxidative stress, inflammation, or injury. The second or multiple hit theory was also proposed for TDP-43 and Fused-In-Sarcoma (FUS) pathogenesis, two proteins that are also linked to FTLD [51]. In contrast to mice used in biomedical research, zebrafish inbred lines are not available. Outbred lines enrich genetic variability and make the animals more resistant and robust, potentially precluding phenotypes. In contrast to mammals zebrafish also have a tremendous regenerative capacity [52]. This could possibly lead to a quick replacement of cells that are not fully functional because of a lysosomal dysfunction and thereby escape detection.

We also cannot exclude that the short Grn1 and Grn2 proteins sharing a similar domain structure might compensate the loss of Grna and Grnb. However, qPCR analysis of the shorter *grn1*/*grn2* did not reveal an upregulation of *grn1*/*grn2* on the transcriptional level in the *grna* ^*−/−*^;*grnb* ^*−/−*^ mutants. Still, we cannot exclude a compensatory upregulation of Grn1/Grn2 in the Grna;Grnb KOs on the translational level. Additional KO of Grn1 and Grn2 can experimentally address this possibility in the future. It is also possible, that the zebrafish Granulins have, despite similar structures, diverse function and are independently regulated. In mice it was shown that Grn and the granulin peptides have opposing functions [53], which might be the case in zebrafish as well.

### KD versus KO phenotypes in zebrafish

Previous KD studies in zebrafish embryos using Grna or Grnb translation inhibition MO resulted in SpMN axonopathies and a reduced number of MPCs [18–20]. In our Grna and Grnb single and double KOs we did not observe extended branching or truncation of SpMN axons despite careful quantifications and controls. Contradictory results obtained from morphants and mutants regarding SpMN axon outgrowth phenotypes have been described previously for other genes related to neurodegenerative diseases, e.g. Tardbp [24, 54] and Fragile X mental retardation 1 (Fmr1) [55, 56]. Similarly, the number of Pax7-positive MPCs in the *grna* ^*−/−*^;*grnb* ^*−/−*^ embryos was not reduced as described after Grna or Grnb KD [20]. We speculate that the reduced number of MPCs in the morphants is due to toxic effects of the MO since the KD embryos have also morphological defects [20], which we do not observe in the KO embryos. To our knowledge, none of the studies in Grn KO mice showed muscle or motor neuron axons phenotype. Most importantly, GRN is not linked to any muscle disease and only published to be a minor risk factor for ALS [57, 58] and therefore not expected to affect SpMN axon outgrowth or muscle development but rather exclusively lead to NCL or FTLD-related phenotypes when mutated. These discrepancies raise concerns about the validity of the MO-induced phenotypes and suggest that MO-induced (neuro)toxicity is responsible for the SpMN axon outgrowth and MPCs phenotypes observed in morphants. With the ease of generating mutations in the zebrafish genome by the novel genome editing tools such as CRISPR/Cas9 we now have additional tools to circumvent the KD technologies [59].

## Conclusion

In summary, we successfully generated Grna and Grnb KO mutants by targeted genome editing using ZFNs. The Grna and Grnb single and double KO mutants did not develop phenotypes previously published in KD studies [18–20, 23], highlighting the importance of generating stable genetic mutations. Moreover, the *grna* ^*−/−*^;*grnb* ^*−/−*^ mutants are also devoid of FTLD-TDP/*GRN* or NCL/*GRN* related neuropathology and biochemical alterations reported for Grn KO mice [1, 5, 11, 24]. Since zebrafish *granulins*, especially *grna*, are upregulated after traumatic injuries [31, 60, 61] or upon infection [62, 63] it is likely that the zebrafish Granulins are involved in the inflammatory response as well as in regeneration in line with their enrichment in haematopoietic tissues [14, 31, 64]. Moreover, it was demonstrated that the inflammatory response in Grn KO mice is exaggerated after an injury [65, 66]. Further clarification of the role of the zebrafish Granulins in injury, inflammation, and regeneration remains to be addressed in more specialized functional assays and might provide further support for the hypothesis that *GRN* mutation carriers need to be exposed to a second hit to develop FTLD-TDP/*GRN*.

## Materials and Methods

### Zebrafish

Zebrafish embryos were kept at 28.5°C and staged according to Kimmel et al. [67]. For all experiments, unless stated otherwise, the wild-type line AB was used. Animal experiments were performed in accordance with the animal protection standards of the Ludwig-Maximilians University Munich and were approved by the government of Upper Bavaria (Regierung von Oberbayern, Munich, Germany, GZ:55.2-1-54-2532-127-10). The transgenic line Tg(*apoeb*:lynEGFP) was used in this study [39]. Embryo, larvae, and adult zebrafish were euthanized by an overdose of Tricaine (300 mg/l, Pharmaq Ltd) [68].

### ZFN and identification of induced genomic lesions

CompoZr Custom zinc finger nucleases (ZFN) (Sigma-Aldrich) were designed for the *grna* (ENSDARG00000004954, Zv8) and the *grnb* (ENSDARG00000025081, Zv8) locus. ZF nucleotide recognition sequences are highlighted in uppercase:

*grna* ZFN: TTTGCTCGCAGTGCCCCAataatGAAGTCTGTGAAGCAGGC
*grnb* ZFN: TACCACCTGCTGCCAGatgcctgATGGGGGCTGGGGCT

ZFN-induced genomic mutations were identified by PCR amplification around the ZFN target site and subsequent restriction endonuclease digest. Samples were derived from euthanized 1dpf or 2dpf embryos or adult fin biopsies from anesthetized adult zebrafish (1xTricaine, 80 mg/l, Pharmaq Ltd). Oligonucleotides used are displayed in 5’-3’ orientation: P_*grna* ZFN forward TTCAGTCATTGTTTCAGAGGTCA, P_*grna* ZFN reverse TTCCTCTGATCCACTTTCTACCA, P_*grnb* ZFN forward AATGACACAAGACGTCCTCATAAA, P_*grnb* ZFN reverse AAAAATAATAACCACAGCGCAACT. 5μl of the 17μl PCR reactions were then digested at 37°C for approx. 3h with the following enzymes: *grna* ZFN PCR: Eco91I (Fermentas), *grnb* ZFN PCR: XcmI (New England Biolabs)

### Whole mount immuno-fluorescence staining

Whole mount immuno-fluorescence staining was performed as previously described by Schmid and colleagues [24]. For znp1 staining 28hpf embryos and for Pax7 staining 24hpf embryos were anesthetized in E3 1xTricaine (80 mg/l, Pharmaq Ltd) and fixed in 4% PFA.

### Neutral red staining

Neutral Red (Sigma-Aldrich) staining was performed at 3dpf to estimate the number of microglia as described by Herbomel and colleagues [33]. Larvae were anesthetized in E3 1xTricaine (80 mg/l, Pharmaq Ltd). After *in vivo* recording larvae were euthanized by an overdose of Tricaine (300 mg/l, Pharmaq Ltd) [68].

### Acridine orange staining

Acridine orange (Sigma-Aldrich) staining was performed at 5dpf as described by Paquet and colleagues [25]. Larvae were anesthetized in E3 1xTricaine (80 mg/l, Pharmaq Ltd). After *in vivo* recording larvae were euthanized by an overdose of Tricaine (300 mg/l, Pharmaq Ltd) [68].

### PTZ treatment and locomotion analysis

Locomotion analysis was performed in a ZebraBox Revolution (ViewPoint). Data was recorded by a digital camera with high resolution 1024x768 at 30 frames/s and analysed by ZebraLab tracking software version 3,22,3,9 (View Point). 5dpf larvae were placed into the 24-well plates (one larvae/well) in 800μl embryo medium (E3), 800μl 1%DMSO (Merck) E3, or 800μl 15mM PTZ (Sigma-Aldrich) 1%DMSO E3. The plate was transferred to the ZebraBox and the following tracking was performed: 30min adaptation, 5min tracking. Inactivity was defined as movements with a velocity below 2mm/s and large movements included all with a velocity above 6mm/s. After recording larvae were euthanized by an overdose of Tricaine (300 mg/l) (Pharmaq Ltd) [68].

### Cloning

For *grna* cDNA (ENSDART00000137973, Zv9) cloning 5dpf AB cDNA and for *grnb* cDNA (ENSDART00000105686, Zv9) cloning 4dpf AB cDNA was used as a template. Oligonucleotides used are displayed in 5’-3’ orientation: P_*grna* forward CTGCTCAAAAAATGTTGAGACTG, P_*grna* reverse GCTCTAGAGCTTATAGAGTTAGGGCTCGTTTC, P_*grna*+MYC reverse GCTCTAGAGCTTATTCATTCAAGTCCTCTTCAGAAATGAGCTTTTGCTCCATCCCTAGAGTTAGGGCTCGTTTC, P_*grnb* forward ATGGTGCGTGCAGCTTTCAT, P_*grnb* reverse TTAGAGAGAATTATTCCACCACGT, P_*grnb*+MYC reverse TTATTCATTCAAGTCCTCTTCAGAAATGAGCTTTTGCTCCATGAGAGAATTATTCCACCACGT. All PCR products were cloned as described by Schmid and colleagues [24].

### mRNA injections

mRNA for microinjections was synthesized in vitro using the MessageMAX T7 mRNA transcription kit (Epicentre) and the mMESSAGE mMACHINE T7 kit (Ambion) according to the manufacturer’s instruction. 2–4pl of 0.4μg/μl ZFN mRNA or 0.2μg/μl *grnb* mRNA (ENSDART00000105686, Zv9) were injected into the yolk. Translation of the mRNAs was confirmed using Western blotting.

### qPCR

Samples for qPCR derived from 5dpf larvae that were anesthetized in E3 1xTricaine (80 mg/l, Pharmaq Ltd) and put in liquid nitrogen or from adult zebrafish biopsies taken from 7mpf or 22mpf zebrafish euthanized by an overdose of Tricaine (300 mg/l, Pharmaq Ltd) [68]. The RNeasy kit (Qiagen) was used with on column DNase I (Qiagen) treatment for total RNA isolation. cDNA synthesis was performed with M-MLV reverse transcriptase (Invitrogen) and Random Primer Mix (NEB), followed by a RNase H (Invitrogen) digest. qPCR was performed using SsoFast Evagreen Supermix (BioRad) with standard protocols. Oligonucleotides used are displayed in 5’-3’ orientation:

P_*actb1* forward GATCTTCACTCCCCTTGTTCA (ENSDARE00000990736, Zv9), P_*actb1* reverse AAAACCGGCTTTGCACATAC (ENSDARE00001086149, Zv9), P_*ctsd* forward GAAATACAACCTGGGCTTCC (ENSDARE00001100358, Zv9), P_*ctsd* reverse GAAGGTCTGGACAGGAGTGC (ENSDARE00000580787, Zv9), P_*flnca* forward CCTTCGTGGGTCAGAAGAAC (ENSDARE00000111992, Zv9), P_*flnca* reverse GGAGTTCTAGGACCGTGGAC (ENSDARE00000408366, Zv9), P_*flncb* forward GGCCCTACAAAGTGGACATC (ENSDARE00000522696, Zv9), P_*flncb* reverse CTTCAAACCAGGCCCATAAG (ENSDARE00000736832, Zv9), P_*grn1/grn2* forward CCACCAGAACCTTCCAGAAA (ENSDARE00000899372, and ENSDARE00000953342, Zv9), P_*grn1/grn2* reverse TGGCCAGTTCTAGTCTTGCAG (ENSDARE00000948245, ENSDARE00001069257, and ENSDARE00000948034, Zv9) P_*grna* qPCR forward ACCACATGGGGATGTTGC (ENSDARE00000060406, Zv9), P_*grna* qPCR reverse CCAAGTCTCCGGCTGAAATA (ENSDARE00000985214, Zv9), P_*grnb* qPCR forward GTCGCAGGAAGCCATTAGAG (ENSDARE00000188550, Zv9), P_*grnb* qPCR reverse CAGCATGTTGTATCTTCTGGACA (ENSDARE00000468841, Zv9), P_*stat3* forward CCAGCTCAAAATCAAAGTGTG (ENSDARE00000257597, Zv9), P_*stat3* reverse TTGGATTCCTCCATGTTCATC (ENSDARE00000257592, Zv9), P_*tbp* forward TCAGCATGGAGCAGAACAAC (ENSDARE00000320694, Zv9), P_*tbp* reverse CCCATACGGCATCATAGGAC (ENSDARE00000327919, Zv9). All samples were run in triplicates and normalized to the housekeeping genes *TATA-binding protein (tbp)* and *actin*, *beta 1 (actb1)*. Relative mRNA abundance was calculated using the ΔΔCt method.

### Antibodies

α-Tubulin, monoclonal, mouse, Sigma-Aldrich Cat# T6199, RRID:AB_477583, Western blotting (WB) 1:10000; Calnexin, polyclonal, rabbit, Enzo Life Sciences Cat# SPA-860D, RRID:AB_2069021, WB 1:10000; Cathepsin D, raised against rat Cathepsin D, polyclonal, rabbit, WB 1:8000 [47]; Pax7, monoclonal, mouse, Developmental Studies Hybridoma Bank Cat# pax7, RRID:AB_528428, immuno-fluorescence (IF) 1:100; znp-1, mouse, Developmental Studies Hybridoma Bank Cat# znp-1, RRID:AB_531910, IF 1:100; Anti-mouse IgG, HRP conjugated (conj.), Promega Cat# W4021, RRID:AB_430834, WB 1:5000; Anti-rabbit IgG, HRP conj., Promega Cat# W4011, RRID:AB_430833, WB 1:10000; goat anti-mouse IgG (H+L) antibody, Alexa Fluor 488, conjugated, Life Technologies Cat# A11001, RRID:AB_10566289, IF 1:500

The following antibodies were generated by the Institute of Molecular Immunology, Helmholtz Centre Munich by standard procedures: Grna 12E7-111 (Grna epitope: KQKKPETQRTTTRPTGTTS, NP_001001949.2), WB 1:1, monoclonal rat IgG2b; Grnb 11F4-11 (Grnb epitope: CTKSSSSTWWNNSL, NP_997903.1), WB 1:1, monoclonal rat IgG2c; Anti-rat IgG2b, HRP conj., WB 1:2000; Anti-rat IgG2c, HRP conj., WB 1:32000. All supernatants of hybridoma pools were first screened ELISA with the respective peptides and subsequently by Western Blotting with respective tagged grn proteins.

### Western blotting

Western Blotting was performed as previously demonstrated by in Schmid and colleagues [24]. Samples for Western Blotting were derived from 1.5dpf embryos or 3dpf or 5dpf larvae that were anesthetized in E3 1xTricaine (80 mg/l, Pharmaq Ltd) and subsequently put in liquid nitrogen or from adult zebrafish biopsies taken from 7mpf or 22mpf zebrafish euthanized by an overdose of Tricaine (300 mg/l, Pharmaq Ltd) [68]. Quantifications were performed with ImageJ.

### Image acquisition and *in vivo* imaging

Images were taken with a Cell Observer CSU-X1 Yokogawa Spinning Disk, AxioCam MRm and Evolve 512 (Zeiss). For in vivo imaging 1.5dpf embryos or 3dpf/5dpf larvae were anesthetized in E3 1xTricaine (80 mg/l) (Pharmaq Ltd) and were mounted in 1.5% agarose (Invitrogen) dissolved in E3 1xTricaine. Embedded embryos were covered with E3 1xTricaine. Brightness and contrast were adjusted using Zen blue (Zeiss) and ImageJ. Image stitching was performed with the ImageJ plugin Image stitching [69] and microglia were tracked with the ImageJ plugin mTrackJ [70].

### Statistics

The software Graph Pad Prism 6 (Graph Pad Software) was used for statistical analyses of the data. The statistical tests used are indicated in the figure legend.

## Supporting Information

S1 Fig The mRNA levels are reduced in all analysed *grna* and *grnb* mutants. **A**: mRNA levels of *grna* in 5dpf old Grna KOs and wildtype. **B**: *grnb* mRNA levels in 5dpf Grnb KOs and age-matched wildtype controls. Normalized to *actb1* and *tbp*. qPCR. S.E.M. Mann-Whitney test (one-tailed). *p < 0.05. n = 3. n = 1 *grna* ^mde54c−/−^ and *grnb* ^mde357a−/−^.
(TIF)

S2 Fig Grna and Grnb single and double KOs have no obvious morphological phenotype. Images of 1.5dpf old wildtype, Grna and Grnb single and double KOs. Anterior to the left. Lateral view. Single images were taken on a spinning disk microscope using transmitted light and were stitched using the Image Stitching plugin of ImageJ.
(TIF)

S3 Fig Grna and Grnb single and double KOs are not developmentally delayed. A: The number of somites in *grna* ^*−/−*^ mutants compared to wildtype. **B**: Analysis of the number of somites in *grnb* ^*−/−*^ mutants and wildtype. **C**: The number of somites in *grna* ^*−/−*^;*grnb* ^*−/−*^ mutants and wildtype. SD. Mann-Whitney test (two-tailed). All n.s. n > = 30.
(TIF)

S4 Fig Grna and Grnb single and double KOs lack compensatory upregulation of other paralogues. **A**: mRNA levels of *grnb* in wildtype and Grna KOs. **B**: *grna* mRNA levels in wildtype and Grnb KOs. **C:** *grn1/grn2* mRNA in Grna;Grnb KOs compared to wildtype. Normalized to *actb1* and *tbp*. qPCR experiments with 5dpf old larvae. S.E.M. Mann-Whitney test (two-tailed). **A**, **B**: n = 3, n = 1 *grna* ^mde54c−/−^ and *grnb* ^mde357a−/−^. **C**: n = 4. **D**: Grnb in wildtype and *grna* ^−/−^ mutants at 3dpf. α-tubulin serves as a loading control. **E**: Grna in adult kidney samples from *grnb* ^−/−^ mutants and wildtype. α-tubulin serves as a loading control.
(TIF)

S5 Fig No SpMN axonopathy in Grna and Grnb single and double KOs that lack maternal *grna* and/or *grnb* mRNA. **A-C**: Quantification of the SpMN axon length in homozygous and heterozygous Grna and Grnb single and double KOs that are devoid of maternal mRNA. The SpMN axon length is measured from the exit point of the spinal cord to the growth cone. Spinal motor neuron axon length of the 5 SpMN axon (1–5) above the end of the yolk extension is determined. **A**: Homozygous and heterozygous Grna KO siblings. n = 30. **B**: Homozygous and heterozygous Grnb KO siblings. n = 30. **D**: Homozygous and heterozygous Grna and Grnb double KO siblings. n = 30. S.E.M. Two-way ANOVA. Bonferroni post-test. all n.s.
(TIF)
