# Supplementary figures and images for: Granulin Knock Out Zebrafish Lack Frontotemporal Lobar Degeneration and Neuronal Ceroid Lipofuscinosis Pathology
Source: PLoS One. 2015 Mar 18;10(3):e0118956. doi: 10.1371/journal.pone.0118956 (PMC4365039; doi:10.1371/journal.pone.0118956)

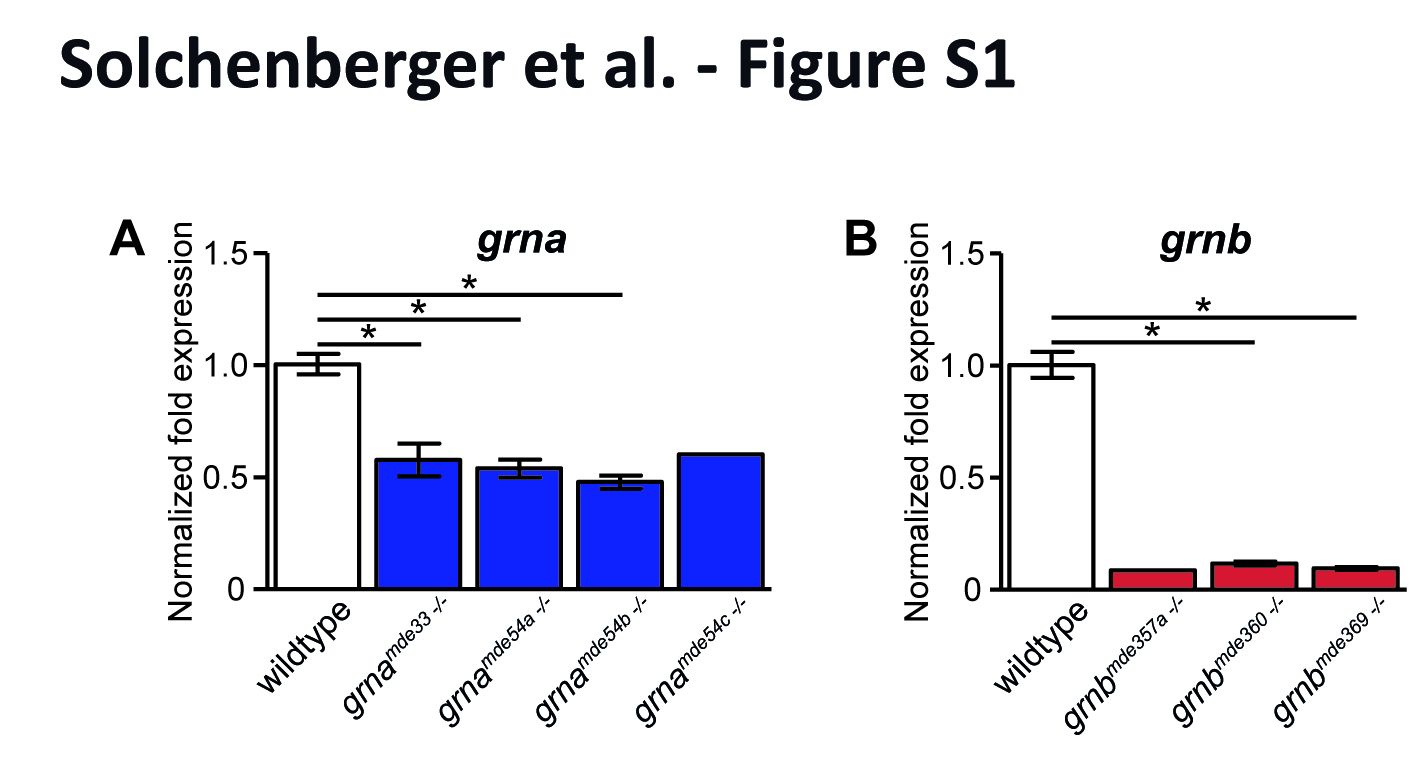

Supplement: S1 Fig — A: mRNA levels of grna in 5dpf old Grna KOs and wildtype. B: grnb mRNA levels in 5dpf Grnb KOs and age-matched wildtype controls. Normalized to actb1 and tbp. qPCR. S.E.M. Mann-Whitney test (one-tailed). *p < 0.05. n = 3. n = 1 grna mde54c−/− and grnb mde357a−/−. (TIF) [file pone.0118956.s001.tif]

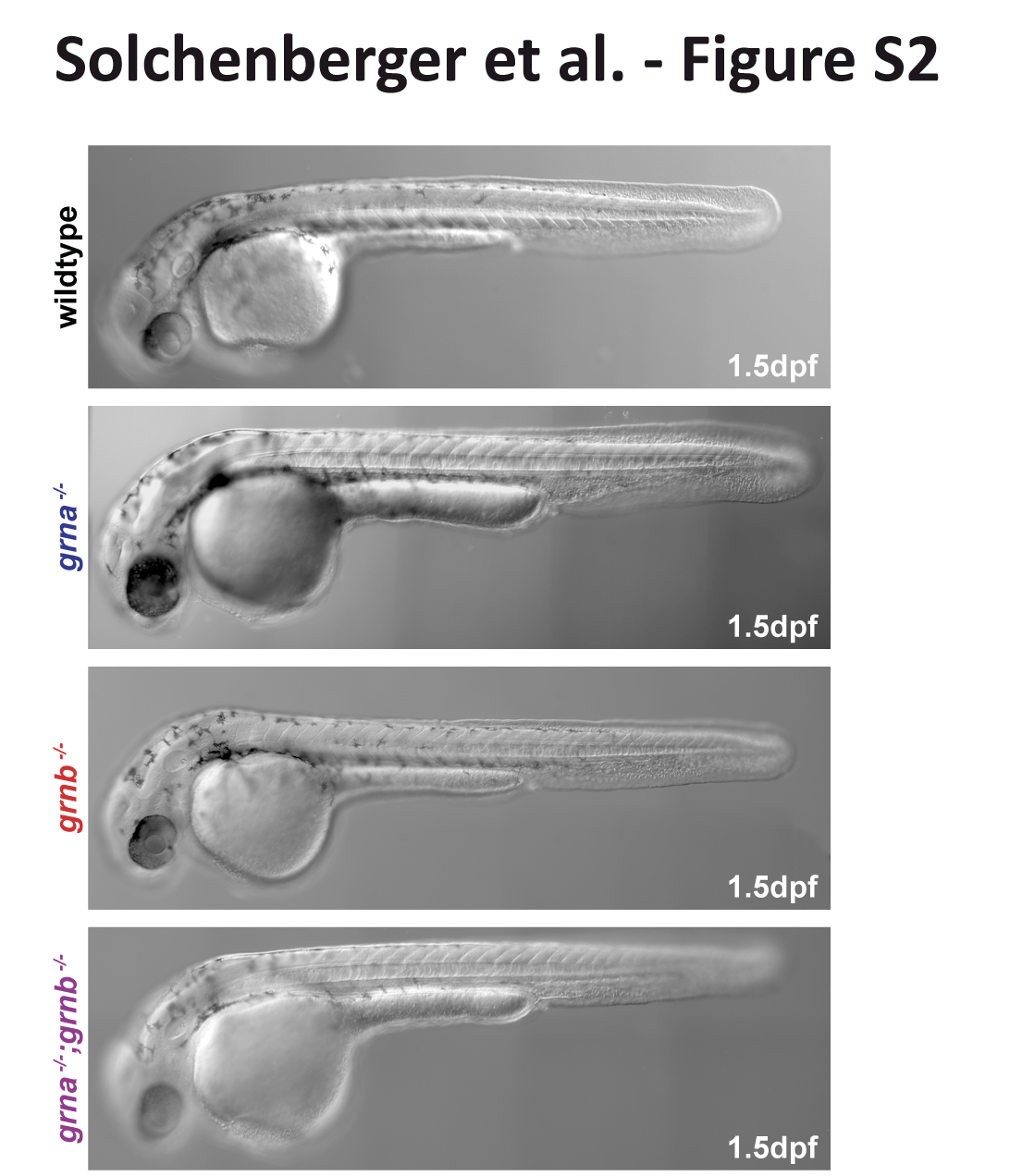

Supplement: S2 Fig — Images of 1.5dpf old wildtype, Grna and Grnb single and double KOs. Anterior to the left. Lateral view. Single images were taken on a spinning disk microscope using transmitted light and were stitched using the Image Stitching plugin of ImageJ. (TIF) [file pone.0118956.s002.tif]

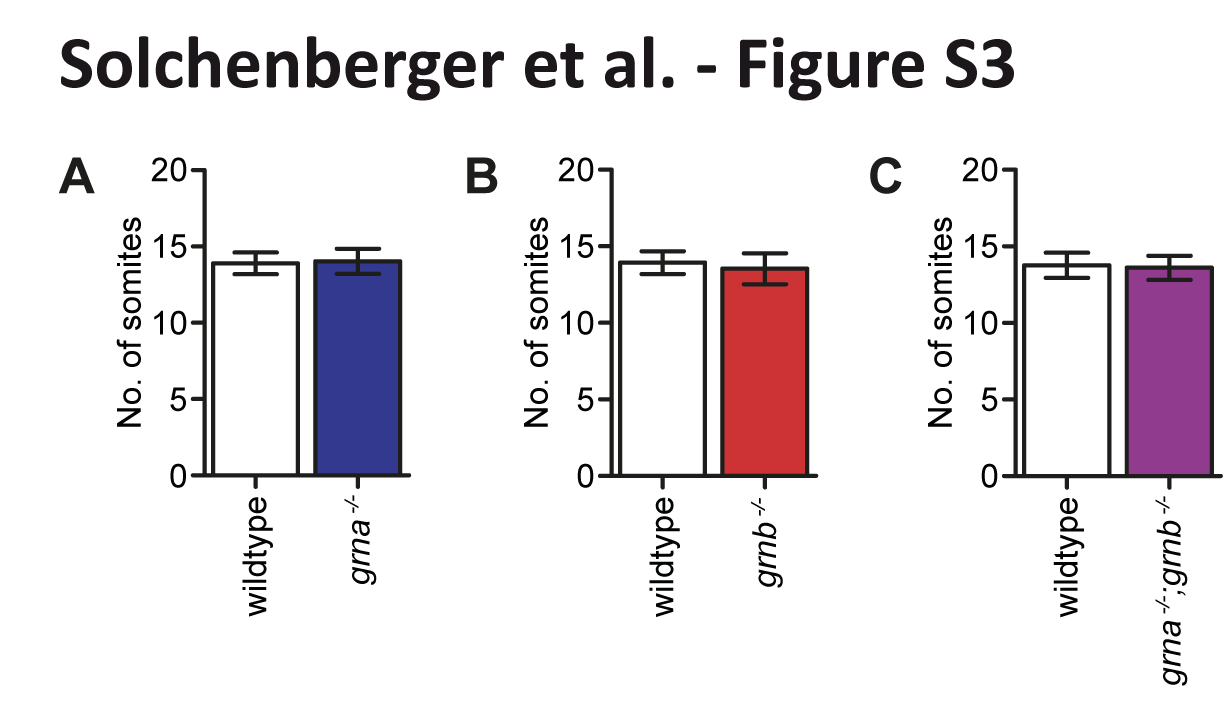

Supplement: S3 Fig — A: The number of somites in grna −/− mutants compared to wildtype. B: Analysis of the number of somites in grnb −/− mutants and wildtype. C: The number of somites in grna −/−;grnb −/− mutants and wildtype. SD. Mann-Whitney test (two-tailed). All n.s. n > = 30. (TIF) [file pone.0118956.s003.tif]

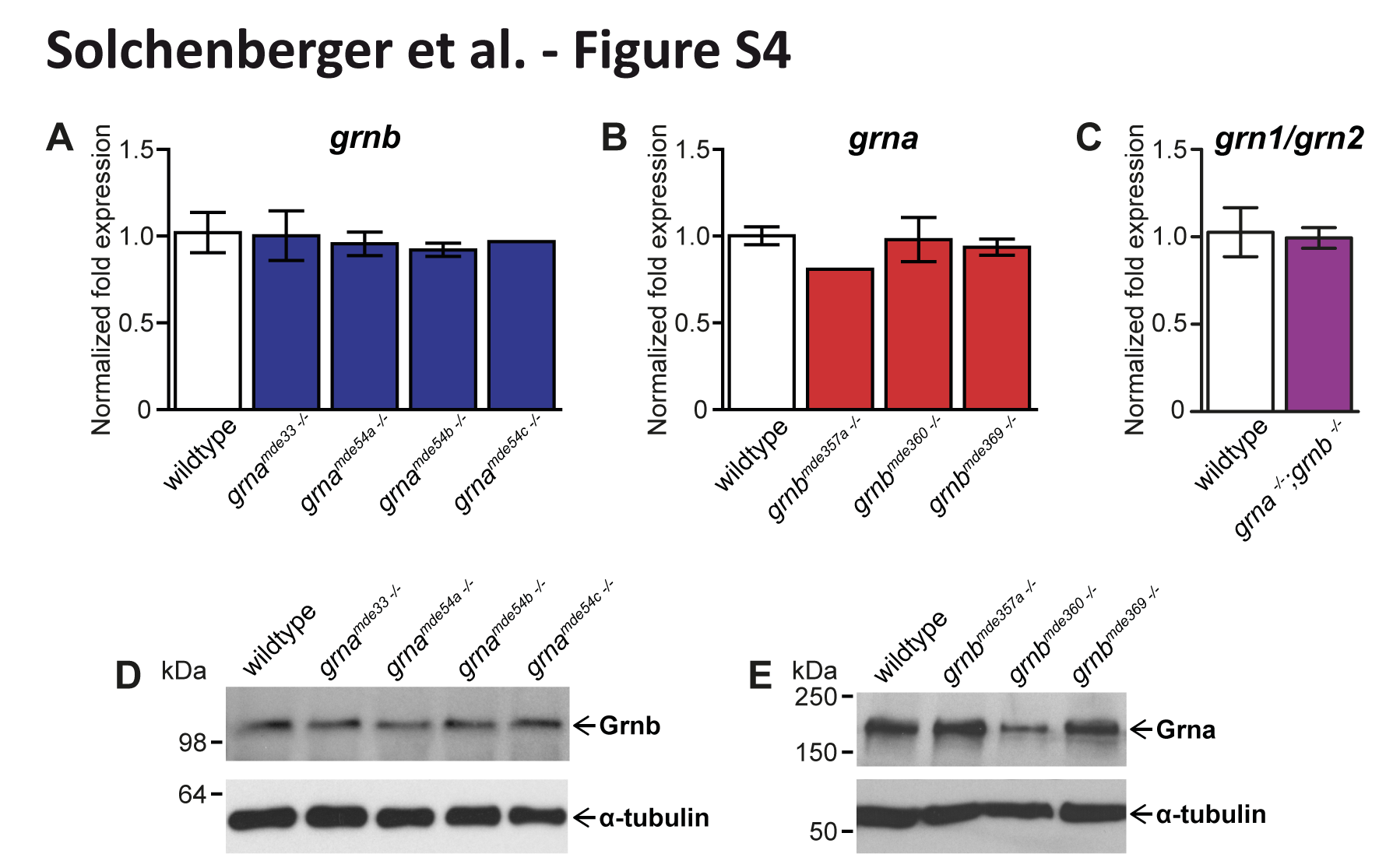

Supplement: S4 Fig — A: mRNA levels of grnb in wildtype and Grna KOs. B: grna mRNA levels in wildtype and Grnb KOs. C: grn1/grn2 mRNA in Grna;Grnb KOs compared to wildtype. Normalized to actb1 and tbp. qPCR experiments with 5dpf old larvae. S.E.M. Mann-Whitney test (two-tailed). A, B: n = 3, n = 1 grna mde54c−/− and grnb mde357a−/−. C: n = 4. D: Grnb in wildtype and grna −/− mutants at 3dpf. α-tubulin serves as a loading control. E: Grna in adult kidney samples from grnb −/− mutants and wildtype. α-tubulin serves as a loading control. (TIF) [file pone.0118956.s004.tif]

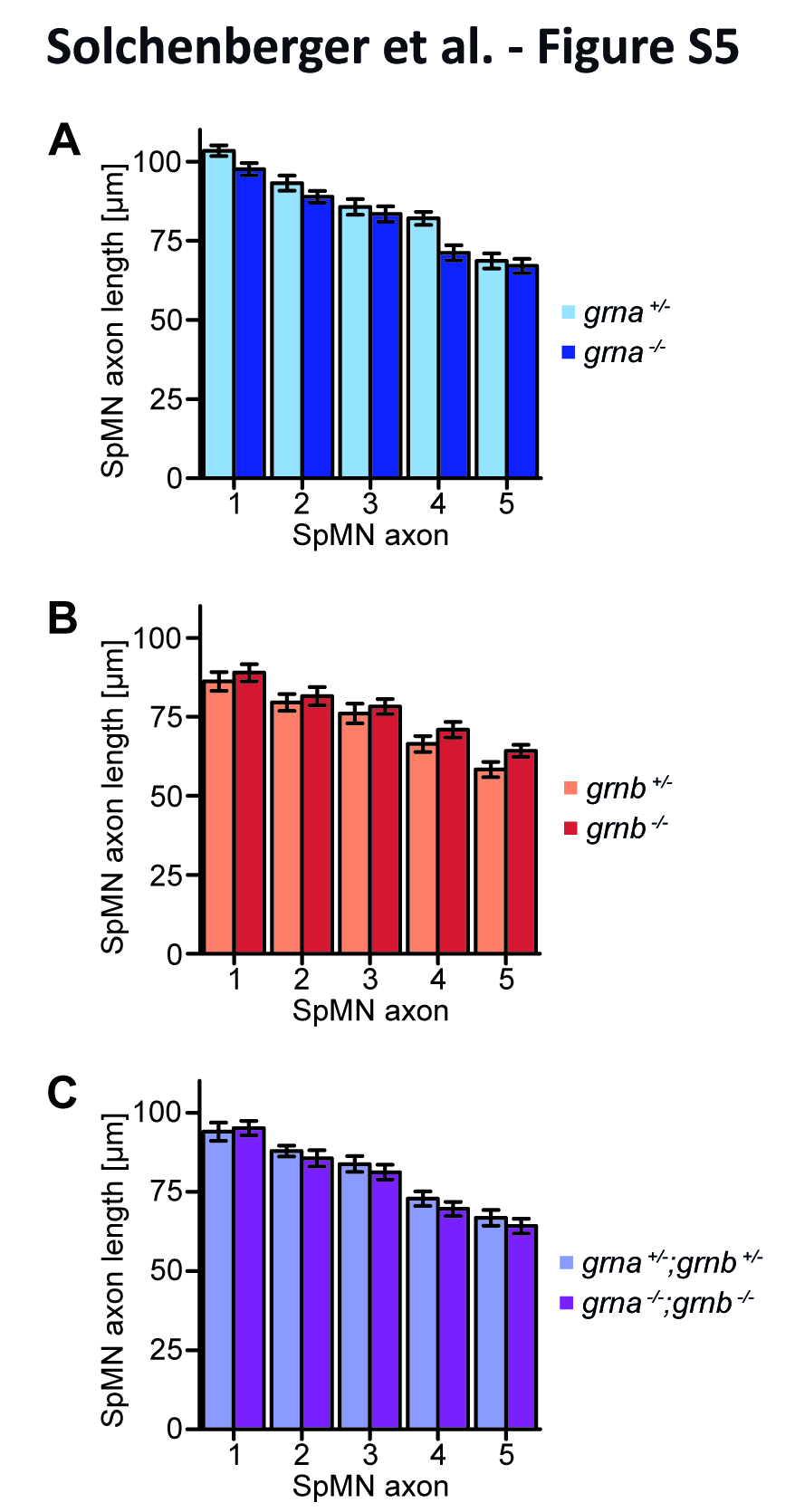

Supplement: S5 Fig — A-C: Quantification of the SpMN axon length in homozygous and heterozygous Grna and Grnb single and double KOs that are devoid of maternal mRNA. The SpMN axon length is measured from the exit point of the spinal cord to the growth cone. Spinal motor neuron axon length of the 5 SpMN axon (1–5) above the end of the yolk extension is determined. A: Homozygous and heterozygous Grna KO siblings. n = 30. B: Homozygous and heterozygous Grnb KO siblings. n = 30. D: Homozygous and heterozygous Grna and Grnb double KO siblings. n = 30. S.E.M. Two-way ANOVA. Bonferroni post-test. all n.s. (TIF) [file pone.0118956.s005.tif]
